# Impact of the COVID-19 pandemic on epidemiological and clinical characteristics of *Mycoplasma pneumoniae* pneumonia in children: a multicenter study from Hubei, China

**DOI:** 10.3389/fped.2024.1388132

**Published:** 2024-10-25

**Authors:** Hui Du, Jun Li, Xilin Li, Junhua Zhao, Wei Lu, Qiong Zhang, Wenchun Liu, Xinbing Luo, Qiao Lu, Sanhong Hu, Jilong Ma, Renzhong He, Bangwu Sha, Lihua Zhang, Jinhui Wu, Junjie Yang, Hongli Li, Hebin Chen, Ying Li, Yang Li, Yaxin Lin, Yuehu Liu, Yabin Wu, Yang Liu, Jianmu Li, Xiaoxia Lu

**Affiliations:** ^1^ Department of Respiratory Medicine, Wuhan Children’s Hospital, Tongji Medical College, Huazhong University of Science and Technology, Wuhan, China; ^2^ Department of Pediatrics, Maternal and Child Health Hospital of Huangshi, Huangshi, China; ^3^ Department of Pediatrics, People’s Hospital of Xishui, Huanggang, China; ^4^ Department of Pediatrics, Xiantao Maternal and Child Health Hospital, Xiantao, China; ^5^ Department of Pediatrics, Yichang Central People’s Hospital, Yichang, China; ^6^ Department of Pediatrics, People’s Hospital of Dangyang, Dangyang, China; ^7^ Department of Pediatrics, The Central Hospital of Enshi Tujia and Miao Autonomous Prefecture, Enshi, China; ^8^ Department of Pediatrics, Taihe Hospital, Hubei University of Medicine, Shiyan, China; ^9^ Department of Pediatrics, Minda Hospital of Hubei Minzu University, Enshi, China; ^10^ Department of Pediatrics, People’s Hospital of Huangpi, Wuhan, China; ^11^ Department of Pediatrics, Affiliated Renhe Hospital of China Three Gorges University, Yichang, China; ^12^ Department of Pediatrics, Huangshi Central Hospital, Huangshi, China; ^13^ Department of Pediatrics, Jingmen Hospital of Traditional Chinese Medicine, Jingmen, China; ^14^ Department of Pediatrics, The Second People’s Hospital of Yichang, The Second Hospital of Three Gorges University Yichang, Yichang, China; ^15^ Department of Pediatrics, Jingmen People’s Hospital/Jingchu University of Technology Affiliated Central Hospital, Jingmen, China; ^16^ Department of Pediatrics, Union Jiangbei Hospital, Huazhong University of Science and Technology, Wuhan, China; ^17^ Department of Pediatrics, Maternal and Child Health Hospital of Hanchuan, Xiaogan, China; ^18^ Department of Pediatric Respiratory Medicine, Hubei Maternal and Child Health Hospital, Wuhan, China; ^19^ Department of Pediatrics, WuHan Asia General Hospital, Wuhan, China; ^20^ Department of Pediatrics, Xiantao First People’s Hospital Affiliated to Yangtze University, Xiantao, China

**Keywords:** *Mycoplasma pneumoniae*, children, prevalence, co-detection, macrolide resistance

## Abstract

**Aims:**

To investigate the epidemiological and clinical characteristics of children with *Mycoplasma pneumoniae* pneumonia (MPP) in Hubei, China.

**Methods:**

We retrospectively analyzed inpatients with MPP from 20 hospitals in Hubei, China from January 2021 to December 2022. The co-detected pathogens of *Mycoplasma pneumoniae* (*M. pneumoniae*) were investigated using targeted next-generation sequencing (tNGS), and 23S rRNA gene mutations were analyzed to assess the macrolide resistance.

**Results:**

*M. pneumoniae* infected 20.7% of patients with CAP, with cough (96.59%) and fever (80.28%) being the most prevalent symptoms. The infection rates in children younger than 1, 1–2, 3–6, 7–12, and older than 12 years were 6.17%, 19.98%, 26.97%, 43.93%, and 2.95%, respectively. Among 1,349 patients undergoing tNGS, the overall co-detection rate was 59.45%, with *Streptococcus pneumoniae* (29.30%), *Haemophilus influenzae* (23.57%), and *Human rhinovirus* (17.21%) being the most commonly co-detected pathogens. In 635 patients undergoing the 23S rRNA gene mutation test, 86.30% exhibited positive mutations (A2063G, 98.00%; A2064G, 1.50%; A2067G, 0.50%). Despite a significant age difference (*P* = 0.037) between macrolide-resistant *M. pneumoniae* and macrolide-sensitive *M. pneumoniae* groups, there were no significant differences in symptoms, lab data, or disease severity.

**Conclusions:**

In Hubei Province, the prevalence of exhibited consistent changes during the COVID-19 pandemic. MPP was prevalent year-round, particularly in summer and autumn, with school-age children being more susceptible. Co-detections of viruses and bacteria were frequent in MPP cases, and macrolide resistance exceeded 85%. Ongoing surveillance of *M. pneumoniae* in children is crucial for understanding the healthcare impact of MPP.

## 1 Introduction

*Mycoplasma pneumoniae (M. pneumoniae)* is a common causative pathogen in community-acquired pneumonia (CAP), accounting for 8%–40% of CAP cases (1–4). *M. pneumoniae* has traditionally been recognized as the predominant pathogen affecting children aged 5–14 years (2, 5). However, there has been a notable rise in reported cases among younger children and newborns (6). The clinical manifestations of *M. pneumoniae* infection are usually mild and self-limiting. In recent years, an increasing number of cases have progressed to severe *M. pneumoniae* pneumonia (SMPP) (7–9) and refractory MPP (RMPP) (10), resulting in multiorgan dysfunction, pleural effusion (11), and serious long-term sequelae, such as bronchiolitis obliterans and bronchiectasis, posing significant challenges for pediatricians.

Previous studies found that *M. pneumoniae* is often co-infected with other pathogens. Compared with *M. pneumoniae* mono-infection, co-infection may lead to more severe inflammatory responses and clinical manifestations, which may prolong or aggravate the course of MPP, suggesting that *M. pneumoniae* co-infection with other pathogens may be one of the causes of SMPP or RMPP (12).

Macrolides are the first-line antimicrobial agents used to treat *M. pneumoniae* infections. However, since 2000, macrolide-resistant strains have become increasingly common, coinciding with the more widespread usage of macrolides (13). More than 85% of *M. pneumoniae* strains among pediatric patients in China have been reported as macrolide-resistant *M. pneumoniae* (MRMP) (14). MRMP infection and excessive immunological inflammation may play important roles in the occurrence and development of RMPP. Therefore, it is essential to dynamically monitor *M. pneumoniae* infections in children and comprehend their epidemiological changes to develop effective preventive measures.

*M. pneumoniae* infection appears as a cyclic epidemic disease worldwide, with intervals of 3–7 years, and can persist for 1–2 years (15). *M. pneumoniae* infection is sporadic throughout the year, with variable high-incidence seasons in different regions. The prevalence of *M. pneumoniae* infection may be influenced by factors such as geography, season, temperature, humidity, and anti-epidemic policies. In this study, we systematically investigated the distribution of MPP in children by age, symptoms, co-detected pathogens, and macrolide resistance. We also provided epidemiological evidence based on a large sample size to shed insights into the prevalence of MPP in central China.

## 2 Materials and methods

### 2.1 Study population

**Figure 1:**
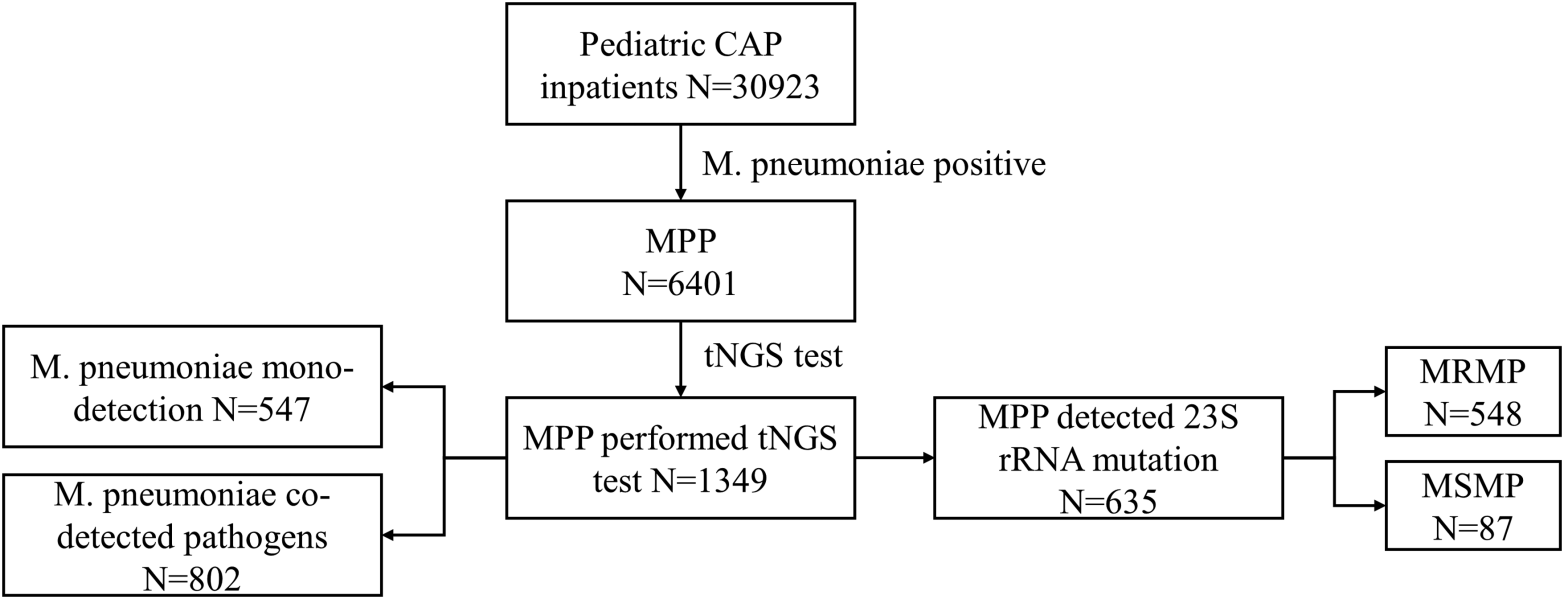
Flowchart of the study population selection. CAP, community-acquired pneumonia; *M. pneumoniae*, *Mycoplasma pneumoniae*; MPP, *Mycoplasma pneumoniae* pneumonia; tNGS, targeted next-generation sequencing; MRMP, macrolide-resistant *Mycoplasma pneumoniae*; MSMP, macrolide-sensitive *Mycoplasma pneumoniae*.

This is a multicenter retrospective study conducted in 20 hospitals across Hubei, China, under the leadership of Wuhan Children's Hospital. The study was approved by the Ethics Committee of Wuhan Children's Hospital (No. 2022R100-E02). We incorporated patients younger than 18 years who were hospitalized with CAP between 1 January 2021 and 30 December 2022. Patient information included demography, symptoms, diagnosis, laboratory measurements, and chest CT scans. We excluded the patients if they were not diagnosed with *M. pneumoniae*-infected pneumonia or if patients with a negative *M. pneumoniae* within 48 h of admission leaving a final cohort of 6,401 inpatients with MPP. To study the distribution of co-detected pathogens, we further analyzed 1,349 patients who underwent targeted next-generation sequencing (tNGS) testing using throat swabs or bronchoalveolar lavage fluid. Of these, 635 patients underwent 23S rRNA gene mutation testing to identify macrolide resistance in *M. pneumoniae* (Figure 1).

The diagnostic criteria for CAP included the following (16): (a) clinical manifestations, including fever, cough, and/or dyspnea; (b) auscultatory findings, such as abnormal breath sounds, wheezes, or crackles; and (c) radiographic evidence, including consolidation, other infiltrates, or pleural effusion. CAP patients with positive results for *M. pneumoniae* DNA or RNA or an *M. pneumoniae* IgG titer ≥160 were considered *M. pneumoniae*-positive. Patients were considered to have *M. pneumoniae* mono-detection if only *M. pneumoniae* were detected. Co-detection was defined as detecting *M. pneumoniae* with at least one additional bacterial or viral pathogen.

### 2.2 Specimen collection

Nasopharyngeal swabs (NS) or bronchoalveolar lavage fluid (BALF) were obtained for *M. pneumoniae* molecular testing. NS samples were collected from the enrolled patients within 24 h of admission. Electronic bronchoscopy was performed when necessary, and BALF samples were collected during these procedures simultaneously within the hospitalization period. Serum samples were collected within 24 h of admission to detect anti-*M. pneumoniae* titers.

### 2.3 tNGS and *M. pneumoniae* 23S rRNA gene mutation detection

The principle of tNGS detection in this study involved multiple polymerase chain reaction (PCR) combined with NGS technology to specifically target the highly conserved region of 198 respiratory pathogens. Specific primers were designed for PCR amplification, which was conducted in an amplification tube to enrich the target pathogens. Subsequently, sequencing joints were connected through a second round of PCR to differentiate the source of the samples. High-throughput sequencing data were obtained using the gene sequencer KM MiniSeqDx-CN, manufactured by Guangzhou Jinquirui Biotechnology Co., Ltd. Bioinformatics software was used to filter the sequencing data and perform a subsequent comparison with the reference genome, thereby enabling the interpretation of pathogen detection outcomes. In addition, the concurrent detection of human DNA within the sample served as a means to monitor the sample's quality.

The tNGS test also encompassed three distinct categories of genetic tests for drug resistance, namely, carbapenem-resistant *Enterobacteriaceae* (CRE), methicillin-resistant *Staphylococcus aureus* (MRSA), and macrolide-resistant *M. pneumoniae*. Point mutations at nucleotide positions A2063, A2064, A2067, and C2617 in domain V of the *M. pneumoniae* 23S rRNA were detected to identify macrolide resistance in *M. pneumoniae*.

### 2.4 Statistical analysis

Statistical analyses were performed using SPSS version 22.0 (International Business Machines Corp., New York, USA) and GraphPad Prism 6.0 (GraphPad Software, San Diego, CA, USA). Continuous variables conforming to a normal distribution were presented as mean ± standard deviation (SD), while those not conforming to a normal distribution were represented by the median. Categorical variables were described as numbers or percentages. Quantitative data were compared using the *t*-test or Mann–Whitney *U*-test. Group comparisons of categorical variables were performed using the chi-square test. A *P*-value of <0.05 indicated statistical significance.

## 3 Results

### 3.1 Monthly distribution of MPP patients

**Figure 2:**
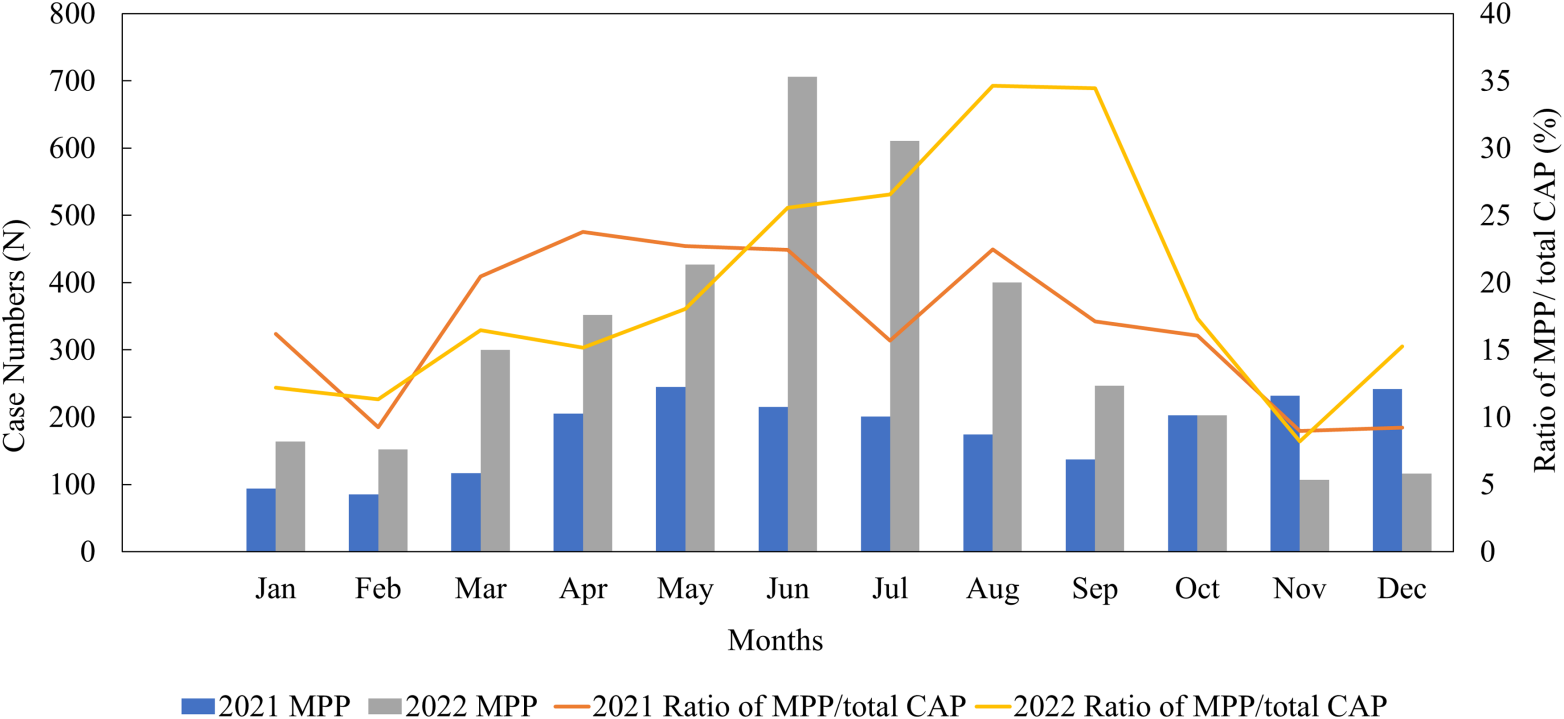
Monthly distribution of MPP patients from January 2021 to December 2022. CAP, community-acquired pneumonia; MPP, *Mycoplasma pneumoniae* pneumonia.

Out of 30,923 CAP inpatients, 6,401 (20.70%) were positive for *M. pneumoniae* in our study. The monthly distribution of all the MPP patients is shown in Figure 2. In 2022, the peak infection rate of MPP occurred between June and September, accounting for approximately 25%–34% of monthly CAP patients. However, in 2021, the MPP spread throughout the year, with no obvious peak (an infection rate of around 17%).

### 3.2 Gender and age distributions of children with MPP

**Figure 3:**
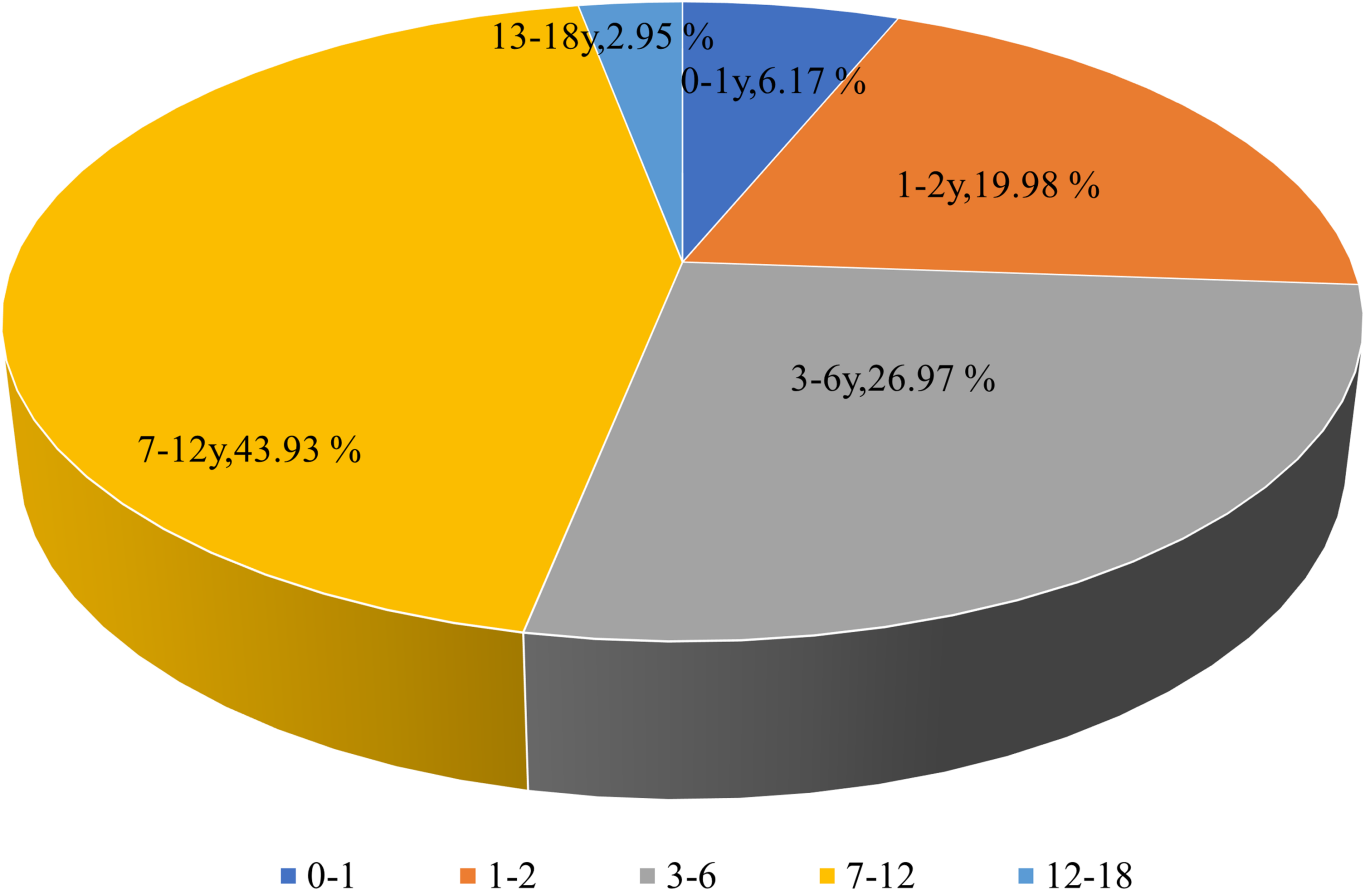
Age distribution of MPP patients.

Among all MPP patients, 2,953 were girls (46.13%) and 3,448 (53.87%) were boys, indicating that the prevalence of MPP was almost evenly distributed in males and females. The median age of children with MPP was 3.79 years. The distribution of MPP patients among children with CAP under 1, 1–2, 3–6, 7–12, and above 12 years was 395 (6.17%), 1,279 (19.98%), 1,726 (26.97%), 2,812 (43.93%), and 189 (2.95%), respectively (Figure 3). Therefore, the MPP rate was higher in older children than in younger ones, with an increasing tendency.

### 3.3 Clinical symptoms of MPP in children

**Figure 4:**
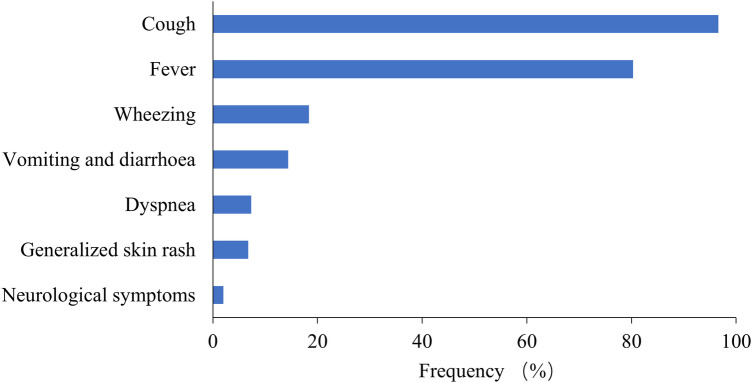
Frequency of common symptoms in children with MPP.

Among the 6,401 MPP patients, the most common symptoms were cough (96.59%) and fever (80.28%), followed by wheezing (18.37%), vomiting and diarrhea (14.37%), and dyspnea (7.31%) (Figure 4). Extrapulmonary manifestations were observed in 562 patients (8.78%), including skin rash in 432 (6.75%) and neurological symptoms in 130 (2.03%). Systemic underlying diseases were present in 487 patients (7.61%), with the most common being recurrent respiratory infection in 447 (6.98%), asthma in 30 (0.47%), congenital heart diseases in 51 (0.80%), bronchopulmonary dysplasia in 27 (0.42%), malnutrition in 18 (0.28%), immunodeficiency diseases in 8 (0.12%), neurological diseases in 38 (0.59%), and malignant tumors in 3 (0.05%).

### 3.4 Co-detected pathogens in children with MPP

**Figure 5:**
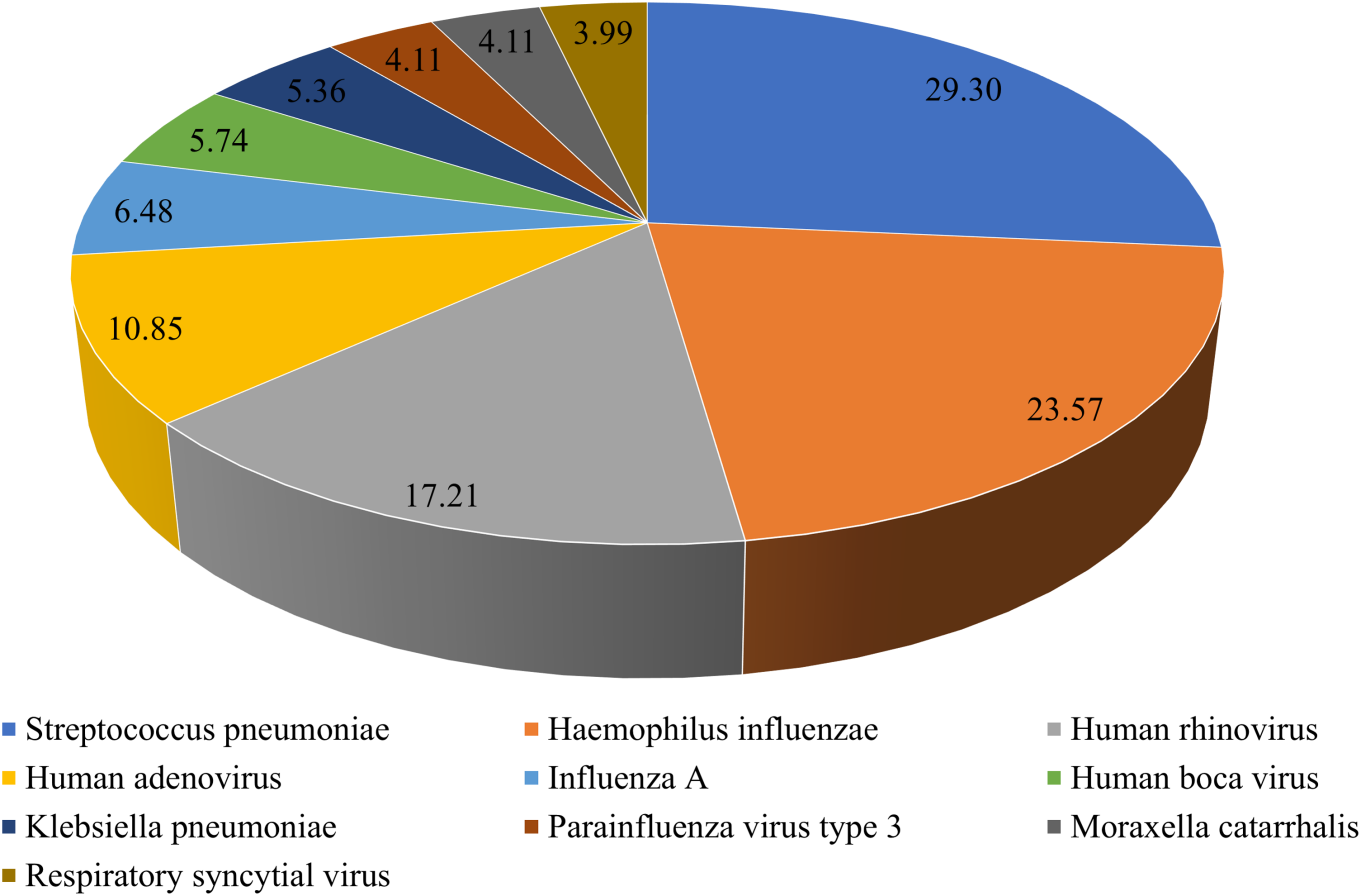
Pathogens detected by tNGS in MPP children.

To understand *M. pneumoniae* co-detection, we analyzed the tNGS results of 1,349 patients. Among them, 547 patients had *M. pneumoniae* mono-detection, while 802 patients had *M. pneumoniae* co-detection, yielding a 59.45% overall co-detection rate. The top 10 co-detected pathogens were *S. pneumoniae* (235, 29.30%), *H. influenzae* (189, 23.57%), human rhinovirus (138, 17.21%), human adenovirus (87, 10.85%), influenza A (52, 6.48%), human boca virus (46, 5.74%), *Klebsiella pneumoniae* (43, 5.36%), parainfluenza virus type 3 (33, 4.11%), *Moraxella catarrhalis* (33, 4.11%), and respiratory syncytial virus (32, 3.99%) (Figure 5).

### 3.5 Macrolide resistance in children with MPP

In total, 635 MPP patients underwent testing for *M. pneumoniae* 23S rRNA gene mutations, including A2063G, A2064G, A2067G, and C2617G mutations. Among these, 548 (86.30%) patients were confirmed to have 23S rRNA mutations, with A2063G being the most prevalent mutation (98.00%), which is consistent with its high prevalence, in Asian countries, followed by A2064G (1.50%) and A2067G (0.50%) (Table 1).

**Table 1:** Statistics of the macrolide-resistant *M. pneumoniae* identified in the 635 patients.

Year	ML-susceptible, *n*	ML-resistant, *n*				ML resistance rate (%)
		A2063G	A2064G	A2067G	C2617G	
2021	32	143	3	1	0	82.12
2022	55	394	5	2	0	87.94
Total	87	537	8	3	0	86.30

ML, macrolide.

### 3.6 Clinical symptoms and laboratory examination findings in MRMP and MSMP children

According to macrolide resistance, the 635 patients were divided into macrolide-sensitive *M. pneumoniae* group (MSMP, *n* = 87) and MRMP group (*n* = 548). The MRMP group was younger than the MSMP group (*P* = 0.037) (Table 2). There were no remarkable differences in clinical symptoms or laboratory test results between the two groups. Although the MRMP group had more severe pneumonia patients than the MSMP group, there was no statistical difference (*P* = 0.154). Furthermore, no significant difference was found in the co-detection rate between the two groups. There was no statistically significant difference between the MSMP group and the MRMP group in terms of the proportion of patients receiving macrolide treatment, nor in the proportion of patients experiencing a fever duration exceeding seven days following the administration of effective antibiotics.

**Table 2:** Comparison of demographic and clinical characteristics of MSMP and MRMP patients.

	MSMP (*n* = 87)	MRMP (*n* = 548)	*P*
Age (y)	6.02 ± 3.05	5.34 ± 2.77	0.037
Gender, male (*n*, %)	46 (52.87)	326 (59.49)	0.245
Underlying disease (*n*, %)	6 (6.90)	40 (7.30)	0.893
Clinical symptoms (*n*, %)			
Fever	70 (80.46)	446 (81.39)	0.837
Cough	82 (94.25)	529 (96.53)	0.300
Wheezing	14 (16.09)	107 (19.53)	0.449
Dyspnea	6 (6.90)	41 (7.48)	0.846
Vomiting and diarrhea	13 (14.94)	79 (14.42)	0.897
Extrapulmonary manifestation	7 (8.05)	47 (8.58)	0.869
Severe MPP (*n*, %)	11 (12.64)	104 (18.98)	0.154
Imaging features (*n*, %)			
Consolidation	52 (59.77)	353 (64.42)	0.402
Pleural effusion	26 (29.89)	176 (32.12)	0.678
Laboratory data			
Leukocyte counts (×10^9^/L)	7.13 (6.07,10.00)	7.78 (5.75,11.67)	0.334
C-reactive protein (mg/L)	16.6 (6.17,40.63)	13.2 (5.29,41.3)	0.891
D-Dimer (mg/L)	0.48 (0.27,1.12)	0.49 (0.29,0.95)	0.824
Co-detection (*n*, %)	54 (62.07)	356 (64.96)	0.549
Macrolide (*n*, %)	87 (100)	486 (88.69)	0.152
Fever ≥7 days after effective antibiotics (*n*, %)	5 (5.75)	38 (6.93)	0.637

MPP, *Mycoplasma pneumoniae* pneumonia; MSMP, macrolide sensitive *M. pneumoniae*; MRMP, macrolide resistant *M. pneumoniae*.

## 4 Discussion

*M. pneumoniae* is a common pathogen that causes CAP in children (2). In the present study, the *M. pneumoniae* infection rate in CAP patients was 20.7%. Although *M. pneumoniae* infection occurs throughout the year, the peak period is usually from the end of summer to the beginning of winter (17, 18). Different from the usual seasonal patterns, our study shows that *M. pneumoniae* infections in Hubei Province remained low in 2021, which was similar respiratory virus trends in Wuhan (19), before peaking in the summer of 2022. During the COVID-19 outbreak in Wuhan, the epicenter, the continuous ongoing use of non-pharmaceutical interventions (NPIs) demonstrated their effectiveness in preventing respiratory infectious diseases, which supports findings from previous studies. However, a prolonged period of reduced exposure to *M. pneumoniae* also leads to an immunity gap (20). We posit that the primary factor contributing to increased susceptibility to *M. pneumoniae* infection is this immunity gap, which is characterized by a decline in humoral immunity. In addition, the *M. pneumoniae* infection rate increased with age, with the highest rate found in school-age children (7–12 years). This trend could be partially explained by the fact that *M. pneumoniae* outbreaks are closely related to close contact among individuals and the population density in kindergartens and schools is relatively high (21).

Consistent with earlier observations (22), our study indicated that the majority of patients exhibited cough (96.59%) and fever (80.28%) as the most common clinical symptoms of MPP. In addition, we discovered that 18.37% of MPP children experienced wheezing. This symptom may be related to the anatomical structure and physiological developmental stage of the respiratory tract in children. We speculate that after *M. pneumoniae* infiltrates the respiratory tract, it may promote the release of inflammatory mediators by inflammatory cells, resulting in inflammatory changes in the respiratory system and increasing airway reactivity, leading to wheezing. Therefore, in children with wheezing episodes, in addition to viral infection, full attention should be paid to the possibility of *M. pneumoniae* infection. The main clinical manifestations of MPP in children are consistent with those observed before the COVID-19 pandemic. However, recent years have seen an increase in RMPP or SMPP cases, which can lead to the prolonging of symptoms such as cough and fever and the aggravation of wheezing and shortness of breath. Multiple factors, including *M. pneumoniae* load, macrolide resistance, systemic inflammatory response, and co-infection, may contribute to the development of SMPP and RMPP (12, 23, 24).

Co-infection with other pathogens is common in MPP patients. In the present study, 59.45% of CAP patients were co-detected with *M. pneumoniae* and other pathogens, which is slightly higher than the earlier recorded rates of 10%–56% (12, 18). This could be attributed to the variations in climate, geographic location, and detection methods. The sensitivity and wide coverage of the tNGS respiratory pathogen panel could contribute to the higher positive rate. We found that the top five co-detected pathogens were *S. pneumoniae*, *H. influenzae*, human rhinovirus, human adenovirus, and influenza A. According to a previous report (18), the most common co-detected viruses in children with MPP were human adenovirus and human rhinovirus. Another study found that the most common bacterial co-infection in children with MPP was *S. pneumoniae*, followed by *H. influenzae* (25), which was consistent with our findings. Interestingly, the nasopharyngeal microbiota of *M. pneumoniae* carriers also had a higher abundance of *H. influenzae* (26). Given the strong association with *H. influenzae*, we recommend including appropriate antibiotic coverage for *H. influenzae* in cases of suspected pneumonia in children. In addition, *M. pneumoniae* carriers were found to have a less diverse microbiota, with an overrepresentation of disease-associated microbiota members compared to non-carriers. However, further research and exploration is needed to understand how *M. pneumoniae* infection affects the evolution of the respiratory bacteriome and virome.

Macrolides are the first-line antibiotics for treating MPP. However, with the increasing prevalence of macrolide resistance in *M. pneumoniae* infections worldwide, especially severe in Asian countries (13, 27), concerns have been raised about the efficacy of macrolides in treating MPP in children. Macrolide resistance is conferred by mutations at several positions in domain V of the 23S rRNA gene, including A2063G, A2064G, A2067G, and C2617G, where A2063G and A2064G mutations confer high-level resistance to all macrolides. Epidemiological studies reported a macrolide resistance rate of 12%–88% among *M. pneumoniae* infections (27, 28). In our study, the prevalence of macrolide resistance was 86.3%, with A2063G being the major mutation. Moreover, we found that children with macrolide-resistant strains were younger than those with macrolide-sensitive ones (Table 2), which contrasts with prior research. To enhance the surveillance of macrolide resistance in *M. pneumoniae*, studies involving a more extensive population are required.

In the present study, we compared macrolide-resistant MPP children to macrolide-sensitive MPP children to reveal whether macrolide resistance affects clinical symptoms, laboratory data, imaging features, and disease severity. We found no significant differences in symptoms, such as fever, cough, and wheezing, macrolides treatment, as well as in laboratory and radiographic findings between MRMP and MSMP groups (Table 2). A study (29) also reported that MRMP and MSMP groups shared similar demographics and clinical characteristics. Therefore, it may be difficult to identify macrolide-resistant cases based on the initial presentations. In addition, there was no difference in disease severity of MPP between the two groups, suggesting that macrolide resistance alone is not a major contributor to the development of severe pneumonia. *M. pneumoniae* infection generally causes mild, and self-limiting disease, raising the question of whether antibiotic treatment is necessary. On the other hand, not only macrolide-sensitive cases but also some macrolide-resistant cases can benefit from macrolide administration due to its immunomodulatory effects. Thus, it remains debatable whether all MPP patients with macrolide resistance should be switched to other anti-mycoplasma drugs. However, alternative antibiotics might be warranted in patients with severe MPP.

There are some limitations to the presented study. As a retrospective clinical study, the techniques used for detecting *M. pneumoniae* were not uniform. In addition, not all MPP patients were tested for macrolide resistance through 23S rRNA gene mutation analysis; therefore, the study population analyzed for macrolide resistance characteristics did not represent the whole population. Further monitoring of macrolide resistance in *M. pneumoniae* is needed in future studies.

## 5 Conclusion

In Hubei Province, the prevalence of *M. pneumoniae* exhibited consistent changes during the COVID-19 pandemic. MPP remained prevalent year-round, particularly in summer and autumn, with school-age children being more susceptible. Co-detections of viruses and bacteria were frequent in MPP cases, and macrolide resistance exceeded 85%. Ongoing surveillance of *M. pneumoniae* in children is crucial for understanding the healthcare impact of MPP.

## Data Availability

The original contributions presented in the study are included in the article/Supplementary Material, further inquiries can be directed to the corresponding authors.
